# Suspected Microplastic-Like Particles Recovered From Paired Human Small Intestine and Large Intestine Specimens: An Exploratory Post-mortem Study

**DOI:** 10.7759/cureus.115390

**Published:** 2026-08-29

**Authors:** Eugen-Toma Radu, Nastaca Alina Palade, Crina Cristina Solomon, Manuela Pia Pumnea, Maria Totan, Claudia Maria Mihuț, Cecilia Georgescu, Claudiu Morgovan, Anca Butuca, Felicia Gabriela Gligor

**Affiliations:** 1 Pathology and Laboratory Medicine, Spitalul Clinic Judetean de Urgenta Sibiu, Sibiu, ROU; 2 Preclinical Department, Faculty of Medicine, Lucian Blaga University of Sibiu, Sibiu, ROU; 3 Biochemistry Department, Faculty of Medicine, Lucian Blaga University of Sibiu, Sibiu, ROU; 4 Biology and Ecology Research Center, Faculty of Sciences, Lucian Blaga University of Sibiu, Sibiu, ROU; 5 Food Industry and Environmental Protection Department, Faculty of Agriculture Sciences, Lucian Blaga University of Sibiu, Sibiu, ROU

**Keywords:** histological inflammation, large intestine, ntestinal specimens, post-mortem study, small intestine, suspected microplastic-like particles

## Abstract

Background

Evidence regarding suspected microplastic (MP)-like particles in human intestinal specimens remains limited. This study evaluated their occurrence and anatomical distribution in paired small and large intestinal specimens.

Methodology

This exploratory post-mortem study included 55 adults examined between March 2022 and December 2025. Intestinal specimens were processed using hydrogen peroxide digestion, glass-fiber filtration, Nile Red staining, fluorescence imaging, and ImageJ-based quantification. These screening methods did not provide polymer-specific identification. Histopathological changes were assessed, and clinical and laboratory data were extracted from medical records.

Results

Suspected MP-like particles were recovered from at least one intestinal specimen in 28 (50.9%) individuals. Detection was numerically more frequent in the large than in small intestinal specimens (38.2% vs. 21.8%), although the paired difference was not statistically significant. Urban residence was less frequent among individuals with screening-positive specimens (p = 0.026). No significant associations were identified for the other clinical and laboratory variables. In exploratory unadjusted analyses, screening positivity co-occurred more frequently with histological inflammation. However, small contingency tables, clinical heterogeneity, and potential confounding preclude causal interpretation. Necrosis was uncommon, and fibrosis was absent.

Conclusions

Suspected MP-like particles were recovered using a non-polymer-specific screening workflow. Their co-occurrence with non-specific inflammation is hypothesis-generating and requires confirmation using polymer-specific methods and rigorous contamination controls.

## Introduction

Human exposure to plastics and microplastics (MPs) is an everyday phenomenon [1,2]. In 2023, global plastic production reached 400.3 million metric tons, underscoring the massive scale of manufacturing and the ubiquitous presence of these materials in contemporary society [3]. Synthesized from natural precursors, plastics undergo complex chemical conversions and physical transformations, primarily through polymerization and polycondensation reactions that assemble core monomers into macromolecular polymer chains [4]. This structural transformation is largely irreversible and requires specialized chemical processing to depolymerize or reconfigure polymer matrices for recycling purposes [5].

Within environmental compartments, particulate plastic debris is conventionally classified by size, predominantly distinguishing between MPs and nanoplastics (NPs). MPs are defined as water-insoluble, synthetic solid particles or polymer matrices of either primary or secondary origin, spanning a size spectrum from 1 μm to 5 mm, irrespective of morphology [6-9]. Although consensus regarding their precise size boundaries remains unsettled, NPs generally refer to particles smaller than 1 μm that arise from the progressive fragmentation of larger debris [10]. Collectively, MP and NP particles constitute the most numerically abundant class of plastic litter worldwide [11].

Based on their provenance, MPs are categorized as primary or secondary particles. Primary particles are intentionally manufactured at microscopic scales for specific industrial or consumer applications, including pre-production resin pellets and abrasive agents such as cosmetic microbeads. By contrast, secondary particles arise predominantly from the photo-oxidative (UV-induced) and mechanical degradation of macroplastic debris. The environmental biodegradability of these polymers is governed by a complex interplay among their raw material feedstocks, macromolecular structure, chemical composition, and ambient factors, including solar radiation, moisture, wind, and microbial activity [7,12,13].

Plastics comprise a highly diverse group of synthetic polymers characterized by distinct chemical configurations, physical properties, and environmental fates. Widely used polymers, such as polyethylene, polypropylene, polystyrene, polyvinyl chloride, and polyethylene terephthalate, dominate global manufacturing and differ markedly in monomeric composition, crystallinity, flexibility, and thermal stability [14]. These intrinsic structural differences influence the fragmentation of macroplastics into MPs and determine their physicochemical properties. Moreover, the incorporation of functional additives, including plasticizers, stabilizers, and colorants, further increases material heterogeneity and may progressively leach into surrounding matrices during environmental weathering [14,15].

In environmental matrices, MPs are rarely chemically inert or pure. Instead, they are typically associated with a heterogeneous mixture of organic molecules and heavy metals. Commercial plastics contain functional additives that are not covalently bound to the polymer matrix and may therefore leach into surrounding ecosystems or biological tissues [16]. Furthermore, the hydrophobic surface chemistry of MPs facilitates the adsorption of ambient environmental contaminants, particularly polycyclic aromatic hydrocarbons, heavy metals, and antibiotics [16-20]. This adsorptive capacity may be significantly amplified by weathering processes, including surface abrasion, microfracture formation, fragmentation, and increased surface roughness. Many of these morphological changes are driven by photo-oxidation, which restructures the polymer at the molecular level and accelerates further degradation [21,22].

Human exposure to MP and NP particles occurs mainly through ingestion and inhalation; inhaled particles may also reach the gastrointestinal tract through mucociliary clearance [23]. Plastic-associated particles or polymer signals have been reported in human feces and several biological tissues [24]. Although these observations suggest potential internal exposure, they do not establish the route of entry, the mechanism of epithelial passage, or subsequent vascular or lymphatic distribution [25]. Interpretation is further limited by heterogeneity in sampling procedures, contamination-control measures, digestion protocols, detection limits, and polymer-identification methods [26].

In gastrointestinal research, recovery of particles from a specimen cannot distinguish among luminal presence, mucosal adherence, incorporation into the intestinal wall, or translocation beyond the epithelium [27]. Moreover, Nile Red fluorescence and birefringence are screening observations that may also arise from biological or other non-plastic materials [28]. Paired assessment of small and large intestinal specimens may therefore provide preliminary information on anatomical distribution, but compartment-specific localization and polymer-specific spectroscopy are required to establish tissue incorporation and polymer identity [29-31].

However, human evidence remains limited, particularly regarding the distribution of suspected MP-like particles across the small and large intestine and their relationship with local histopathological findings. Studies examining paired small and large intestinal specimens from the same individuals are scarce. Against this background, the primary descriptive objective was to determine the proportion of individuals from whom suspected MP-like particles were recovered from either small or large intestinal specimens. Secondary exploratory objectives were to describe their distribution across paired intestinal segments and examine their unadjusted co-occurrence with histopathological alterations and selected demographic, clinical, and laboratory variables. All secondary analyses were considered hypothesis-generating.

## Materials and methods

Study population

This exploratory, post-mortem, observational study included a non-consecutive convenience sample of 55 adults of both sexes, aged 21-94 years, who underwent autopsy at Sibiu County Emergency Clinical Hospital, Romania, between March 2022 and December 2025. Cases were not randomly selected but were included based on the availability of suitable paired small and large intestinal tissue specimens and corresponding clinical data. Tissue collection was performed according to a predefined protocol following ethical approval, whereas clinical and laboratory data were retrospectively retrieved from hospital medical records. Paired tissue specimens from the small and large intestine were collected during routine autopsy procedures within 24 hours after death. Cases were included when paired specimens from both intestinal segments could be collected and processed in accordance with the study protocol. No additional clinical or histopathological eligibility criteria were applied. Cases for which paired specimens could not be obtained or processed according to the protocol were not included. Because a prospective screening log was not maintained, the research database contained information only for the 55 included cases and did not record the total number of autopsies performed at the institution or the cases considered before specimen collection. Therefore, the total number of autopsies and the number of cases not included cannot be reported. The study was approved by the Ethics Committee of Lucian Blaga University of Sibiu (approval number: 10/20.07.2021).

Written informed consent for the research use of post-mortem tissue specimens and associated clinical data was obtained from the next of kin of all included individuals. Information on dietary habits, occupational and environmental exposure and lifestyle factors, recent gastrointestinal procedures, and nutritional support was not systematically available because of the retrospective post-mortem design.

Data sources and variables

Clinical and laboratory data were retrieved from hospital medical records. The recorded baseline characteristics comprised age, sex, area of residence (urban or rural), presence of neoplastic disease, and history of radiotherapy and/or chemotherapy exposure. Laboratory data included the most recent biological results available before death. These included blood glucose, liver biomarkers (alanine aminotransferase, aspartate aminotransferase), pancreatic biomarkers (amylase, lipase), renal function parameters (creatinine, urea, estimated glomerular filtration rate), electrolytes (sodium, potassium, chloride), inflammatory biomarkers (C-reactive protein, procalcitonin), and hematological indices (leukocytes, erythrocytes, platelets, hemoglobin, hematocrit). Certain cardiovascular and pulmonary diagnoses were also recorded from the medical records, including atherosclerosis, chronic ischemic heart disease, myocardial infarction, and bronchopneumonia. These diagnoses were selected as documented cardiopulmonary conditions available in the medical records and were included for exploratory assessment of their potential association with suspected intestinal MP-like particle detection. Diagnoses were coded as binary variables according to their presence or absence.

Intestinal tissue sample collection for histological analysis

Before sampling, the intestinal lumen was opened, luminal contents were removed, and the mucosal surface was rinsed thoroughly with ultrapure water. Small intestinal specimens were collected from the ileum, whereas large intestinal specimens were collected from the sigmoid colon or rectum. These sites were used consistently for paired anatomical assessment; the digestion-based workflow was interpreted at the specimen level and could not establish localization within a specific wall compartment.

Full-thickness intestinal wall specimens were obtained for histological examination. The choice of ileal and distal colonic sites was based on their distinct anatomical and transit characteristics; however, the study was not designed to demonstrate preferential uptake, penetration, or long-term accumulation at either site.

After dissection, three tissue fragments of approximately 1 cm³ each were collected from the region of interest and fixed in 30 mL of Davidson’s solution in capped 50 mL tubes. Davidson’s solution consisted of 2% formaldehyde solution (37-40%), 35% absolute ethyl alcohol, 10% glacial acetic acid, and 53% distilled water, as previously described [32]. To minimize the risk of contamination, only instruments without plastic components were used. Each sample was labeled to ensure traceability and to allow correlation with the corresponding medical records.

Sample preparation for suspected microplastic-like particle analysis

For suspected MP-like particle screening, approximately 5 g of small and large intestinal tissue was collected in sterile 50 mL glass tubes with aluminum caps. Plastic instruments and materials containing plastic components were avoided throughout collection and processing. Before extraction, laboratory instruments were rinsed with acetone. Samples were stored at −80°C until analysis.

Suspected microplastic-like particle identification and quantification

Laboratory personnel wore cotton coats, and extractions were performed in a microbiological hood in an isolated environment without active air circulation. Glass equipment was used during sample handling and extraction, and the glass tubes were covered with aluminum foil during incubation to minimize the risk of contamination.

For each intestinal segment, 1 g of tissue was taken from the approximately 5 g specimen previously collected and transferred to a 50 mL glass tube, where it was mixed with 20 mL of 30% hydrogen peroxide. Tubes were covered with aluminum foil and incubated for seven days at 40°C with periodic vortexing. After cooling to room temperature, samples were centrifuged at 2,000 rpm for two minutes. Relative centrifugal force could not be calculated retrospectively because the rotor radius was not recorded. The supernatant was filtered through a 1 μm glass-fiber filter under vacuum. Filters were dried at 40°C, treated with an additional 3 mL of hydrogen peroxide at 40°C to reduce residual organic material, rinsed with 40 mL of ultrapure water, and dried at 40°C.

Approximately 1 mL of Nile Red solution (0.001 mg/mL) was applied to cover the filter surface, followed by incubation for 15 minutes at room temperature. Filters were rinsed with 60 mL of ultrapure water and dried overnight at 40°C. Fluorescence images were acquired using a transilluminator with standardized camera settings (ISO 200, aperture f/6.3, exposure 1/2 seconds, focal length 45 mm). Nile Red fluorescence was used only for screening and was not considered polymer-specific evidence.

One procedural reagent blank was processed in parallel with each analytical batch through the complete digestion, filtration, staining, imaging, and image analysis workflow. No candidate structures meeting the study criteria were detected in these blanks; therefore, no numerical blank correction was applied. Positive recovery controls were prepared by adding a known number of acrylonitrile butadiene styrene (ABS) particles to blank samples before processing. Recovery was calculated as (particles recovered/particles added) × 100 and ranged from 80% to 110%. Because recovery testing was restricted to ABS particles, it represents only partial validation and does not establish recovery across other polymers, sizes, or morphologies. Separate airborne deposition, filter-only, transport, autopsy room, and histology processing blanks were not performed. In particular, histology processing blanks were not included because the histological workflow was originally designed for conventional histopathological assessment rather than polymer-specific microplastic analysis. Consequently, potential contamination introduced during these processing stages cannot be quantified or excluded.

Suspected MP-like particles were quantified using ImageJ software version 1.54p according to a methodology based on Dong et al. (2023) [33]. Particle burden was defined as the number of suspected MP-like particles detected per gram of intestinal tissue (particles/g). Because 1 g of tissue from each intestinal segment was processed for particle analysis, the number of retained candidate particles corresponded directly to the particle burden expressed as particles/g for that specimen. Because the analytical workflow relied on fluorescence staining, image analysis, and morphological assessment, without polymer-specific spectroscopic confirmation, the detected structures are referred to throughout the manuscript as suspected MP-like particles.

The entire filter surface was examined systematically. Fluorescence images were inspected before and after automated analysis. Candidate structures were assessed according to shape, defined margins, fluorescence intensity, optical appearance, and color. Automatically detected objects were manually reviewed to exclude residual Nile Red deposits and repetitive or non-specific fluorescent artefacts. Only structures satisfying both the predefined morphological criteria and post-processing verification were retained.

To reduce background noise and minimize false-positive detection, an operational minimum size threshold was applied to the minimum Feret diameter (minFeret). Following spatial calibration, the image scale was expressed as P pixels/mm. The minimum accepted dimension was set at three pixels. The corresponding physical threshold was therefore calculated as 3/P mm, yielding 0.05621 mm (56.21 μm). Candidate structures with a minFeret below 56.21 μm were excluded. Structures immediately above this threshold were also excluded when manual review revealed repetitive morphology or optical characteristics consistent with background artefacts. This value represents an operational image analysis threshold exclusively for the fluorescence-based filter image screening workflow, not a polymer-specific analytical limit of detection. It was not applied to the separate polarized light histology images, which were examined descriptively.

The present analytical workflow was designed as a screening approach for detecting suspected MP-like particles based on fluorescence and morphological characteristics. Although this methodology enables standardized particle detection and quantification, it does not provide polymer-specific chemical identification. Definitive confirmation of particle composition would therefore require complementary analytical techniques such as micro-Fourier transform infrared spectroscopy (FTIR), Raman spectroscopy, pyrolysis-gas chromatography/mass spectrometry, or an equivalent polymer-specific method.

Histopathological assessment

Intestinal structural changes, including inflammation, fibrosis, and necrosis, were evaluated in hematoxylin and eosin-stained sections [34]. Foreign body giant cell reaction was not included as a predefined histopathological variable and was therefore not systematically evaluated across all specimens. Slides were examined under unpolarized light using a Leica DM3000 microscope.

Hematoxylin and eosin-stained sections were additionally examined under polarized light as a complementary morphological screening approach. Birefringence does not establish polymer identity because biological and non-plastic exogenous materials may show similar optical behavior. Accordingly, visualized objects were classified as suspected MP-like structures, and no polymer type was assigned [35]. Polymer-specific confirmation by microFTIR, Raman microscopy, or an equivalent technique was not available. Images were acquired using a Leica DM2500 microscope equipped with polarized light filters and a camera.

All histopathological slides were evaluated independently by two pathologists who were blinded to the fluorescence-based intestinal particle screening results. The histopathological assessment considered chronic inflammatory infiltrates, neutrophils in the lamina propria, neutrophils in the epithelium, and mucosal erosion or ulceration, corresponding to the domains included in the Robarts Histopathology Index (RHI). Importantly, the chronic and active inflammatory components were evaluated separately. No acute inflammatory activity was identified in the examined specimens: neutrophils were absent from both the lamina propria and epithelium, and no inflammation-related mucosal erosions or ulcerations were observed. Consequently, the inflammatory findings included in the statistical analysis represented chronic inflammation, defined by lymphoplasmacytic infiltration of the lamina propria, with associated architectural alterations where present. For statistical analysis, chronic inflammation was dichotomized as absent or present.

Agreement between the two independent assessments was substantial (Cohen’s κ = 0.85). Discrepancies were resolved through joint review and consensus. Thus, the initial evaluations were independent and blinded, whereas the final values entered into the study database represented the consensus assessments.

To distinguish chronic inflammation from post-mortem autolysis, the pathologists evaluated viable cellular and architectural features. Chronic inflammation was strictly defined by dense lymphoplasmacytic infiltration of the lamina propria, accompanied by structural tissue remodeling, such as crypt distortion or villous atrophy. By contrast, post-mortem autolysis was identified by diffuse epithelial desquamation, pale staining, and nuclear degenerative changes (pyknosis, karyorrhexis, and karyolysis) in the absolute absence of an associated leukocytic tissue response or vascular margination. Because the RHI evaluates active cellular infiltration, areas displaying exclusively autolytic changes without a host immune reaction were excluded from the inflammatory grading. Underlying gastrointestinal disorders were not used as specific exclusion criteria; therefore, inflammatory histological findings may also reflect pre-existing gastrointestinal disease and were not considered specific to suspected MP-like particle exposure.

Size estimation of suspected microplastic-like structures

The size of suspected MP-like particles was estimated using ImageJ software, following a methodology based on Dong et al. (2023) [33], together with polarized light microscopy analysis. Measurements were performed on acquired microscopic images after calibration of the image scale. The results were expressed in micrometers (µm) and were used only for morphological description, not for polymer identification.

Statistical analysis

Statistical analysis was performed using SPSS Statistics version 26.0 (IBM Corp., Armonk, NY, USA). Graphical representations, including forest plots, were generated using Python version 3.12.13. The distribution of continuous variables was evaluated using the Shapiro-Wilk test and graphical inspection. Normally distributed continuous variables were expressed as mean ± standard deviation, while non-normally distributed variables were expressed as median and interquartile range. Categorical variables were presented as absolute and relative frequencies, n (%).

Comparisons between individuals with and without detectable intestinal suspected MP-like particles were performed using the independent samples t-test for normally distributed continuous variables, the Mann-Whitney U test for non-normally distributed continuous variables, and Fisher’s exact test for categorical variables. Because paired small and large intestinal specimens were obtained from the same individuals, the exact McNemar test was used to compare the paired detection frequency of suspected MP-like particles between the two intestinal segments. The Wilcoxon signed-rank test was used to compare suspected MP-like particle burden between paired small and large intestinal specimens. Associations between local histopathological alterations and suspected intestinal MP-like particle detection were evaluated using Fisher’s exact test. Odds ratios and 95% confidence intervals were calculated from 2 × 2 contingency tables. Additional analyses were performed to evaluate the association between selected cardiovascular and pulmonary diagnoses and suspected intestinal MP-like particle detection. A two-tailed p-value <0.05 was considered statistically significant.

## Results

Study population characteristics according to suspected intestinal microplastic-like particle detection status

Suspected intestinal MP-like particles were identified in 28 of the 55 included individuals (50.9%), whereas 27 (49.1%) individuals showed no detectable suspected MP-like particles in either intestinal segment. Baseline demographic, clinical, biochemical, and hematological characteristics according to intestinal suspected MP-like particles detection status are presented in Table 1. Blood laboratory results were obtained during the terminal hospitalization and represented the last available measurements before death. Availability varied by test; no imputation was performed, and each analysis used complete observations for the respective variable.

**Table 1:** Demographic, clinical, biochemical, and hematological characteristics of individuals with and without suspected MP-like particles detected in intestinal specimens. Data are presented as mean ± standard deviation, median (interquartile range), or n (%) as appropriate. Laboratory variables represent the last available measurement during the terminal hospitalization before death. Variable-specific missing data were not imputed, and analyses were based on available observations for each variable. MPs = microplastics; DBIL = direct bilirubin; TBIL = total bilirubin; AST = aspartate aminotransferase; ALT = alanine aminotransferase; eGFR = estimated glomerular filtration rate; CRP = C-reactive protein

Variable	Overall (n = 55)	No suspected MP-like particles detected (n = 27)	Suspected MP-like particles detected (n = 28)	Test statistic	P-value
Demographic characteristics					
Age, years	68.62 ± 15.35	68.37 ± 14.92	68.86 ± 16.03	*t* = −0.116	0.908
Male sex, n (%)	29 (52.7)	18 (66.7)	11 (39.3)	—	0.060
Urban residence, n (%)	34 (61.8)	21 (77.8)	13 (46.4)	—	0.026
Clinical characteristics					
Neoplasm, n (%)	14 (25.5)	10 (37.0)	4 (14.3)	—	0.068
Radiotherapy and/or chemotherapy, n (%)	8 (14.5)	5 (18.5)	3 (10.7)	—	0.469
Blood glucose, mg/dL	132.00 (102.00, 195.00)	129.00 (102.00, 205.00)	135.50 (96.75, 172.50)	*U* = 353.5	0.680
Liver biomarkers					
DBIL, mg/dL	0.94 (0.81, 1.30)	0.94 (0.86, 0.97)	0.97 (0.79, 1.45)	*U* = 15.0	0.734
TBIL, mg/dL	0.81 (0.56, 1.19)	0.70 (0.51, 1.03)	1.02 (0.68, 1.50)	*U* = 273.5	0.078
AST, U/L	41.00 (28.50, 103.50)	41.00 (30.50, 96.50)	35.00 (23.50, 139.50)	*U* = 353.5	0.680
ALT, U/L	26.00 (16.00, 49.00)	28.00 (15.50, 47.50)	23.00 (16.00, 70.50)	*U* = 357.0	0.723
Pancreatic biomarkers					
Amylase, U/L	44.00 (33.00, 82.00)	43.00 (11.00, 87.00)	44.00 (33.00, 82.00)	*U* = 296.5	0.170
Lipase, U/L	25.00 (15.00, 105.00)	87.00 (33.50, 129.50)	23.50 (15.00, 44.00)	*U* = 16.0	0.233
Renal biomarkers					
Urea, mg/dL	54.00 (40.50, 89.00)	55.00 (39.50, 89.50)	53.00 (43.50, 87.50)	*U* = 370.0	0.893
Creatinine, mg/dL	1.26 (0.80, 2.06)	1.26 (1.07, 2.07)	1.11 (0.73, 1.92)	*U* = 303.0	0.207
eGFR, mL/minute/1.73 m²	58.80 ± 35.12	54.48 ± 32.88	62.96 ± 37.28	*t* = −0.894	0.375
Electrolytes					
Sodium, mmol/L	139.00 (136.00, 143.50)	140.00 (136.00, 143.50)	139.00 (135.50, 143.00)	*U* = 357.5	0.729
Potassium, mmol/L	4.29 ± 0.87	4.39 ± 0.83	4.20 ± 0.91	*t* = 0.823	0.414
Chloride, mmol/L	103.40 ± 7.56	102.55 ± 8.21	104.22 ± 6.94	*t* = −0.816	0.418
Inflammatory biomarkers					
CRP, mg/L	138.69 ± 93.32	117.43 ± 95.00	154.64 ± 95.12	*t* = −1.105	0.274
Procalcitonin, ng/mL	1.58 (0.32, 2.56)	0.33 (0.23, 1.74)	1.80 (1.02, 17.98)	*U* = 12.5	0.181
Hematological biomarkers					
Leukocytes, ×10³/µL	12.64 (8.03, 17.71)	11.37 (8.03, 17.42)	13.49 (8.21, 18.69)	*U* = 348.0	0.614
Erythrocytes, ×10⁶/µL	3.93 ± 1.05	4.10 ± 0.97	3.78 ± 1.12	*t* = 1.144	0.258
Platelets, ×10³/µL	218.00 (132.00, 313.00)	218.00 (170.50, 316.00)	216.50 (119.50, 304.00)	*U* = 347.5	0.608
Hemoglobin, g/dL	11.80 ± 3.09	12.56 ± 2.84	11.07 ± 3.20	*t* = 1.832	0.073
Hematocrit, %	36.00 ± 9.29	37.81 ± 8.79	34.27 ± 9.58	*t* = 1.425	0.160

Overall, no statistically significant differences were observed between the two groups in age, sex, neoplasm prevalence, previous radiotherapy and/or chemotherapy, liver biomarkers, renal function parameters, inflammatory markers, or hematological indices. The only statistically significant difference was observed for residence area. Urban residence was less frequent among individuals with suspected intestinal MP-like particles compared with those without detectable intestinal MP-like particles (13/28 (46.4%) vs. 21/27 (77.8%), p = 0.026). Given the exploratory design and the numerous comparisons performed across the demographic, clinical, and laboratory variables presented in Table 1, this isolated finding should be interpreted cautiously.

Anatomical distribution and paired comparison of suspected intestinal microplastic-like particles

Suspected intestinal MP-like particles were identified in 28 of the 55 included individuals (Table 2). With regard to anatomical site, suspected MP-like particles were detected in the small intestine in 12 (21.8%) cases and in the large intestine in 21 (38.2%) cases. Five (9.1%) individuals showed suspected MP-like particles in both intestinal segments, whereas 7 (12.7%) individuals were positive only in the small intestine and 16 (29.1%) individuals only in the large intestine. No suspected intestinal MP-like particles were detected in 27 (49.1%) individuals. Because small and large intestine samples were obtained from the same individuals, a paired analysis was performed. Suspected MP-like particles were numerically more frequently detected in the large intestine than in the small intestine; however, the difference did not reach statistical significance using the exact McNemar test (p = 0.093). Among specimens with detected suspected MP-like particles, the median particle burden was 1.0 (IQR = 0.98-1.93) particles/g in the small intestine (n = 12) and 1.0 (IQR = 1.0-1.9) particles/g in the large intestine (n = 21). In the paired analysis of all 55 cases, including specimens with no detected particles, particle burden did not differ significantly between the two intestinal segments (Wilcoxon signed-rank test, p = 0.144). Overall, large intestine-only detection represented the most frequent positive pattern, but the paired analysis did not confirm a statistically significant predominance of suspected MP-like particle detection in the large intestine in this cohort.

**Table 2:** Anatomical distribution of suspected intestinal MP-like particles in the study population (N = 55). Data are presented as n (%), with percentages calculated using the total study population (N = 55) as the denominator. *: Segment-specific categories are not mutually exclusive because individuals with detection in both intestinal segments are included in both the small and large intestine categories. ^†^: Mutually exclusive detection patterns sum to the 28 individuals with suspected MP-like particles detected in at least one intestinal segment. MP = microplastic

Detection category	n (%)
Overall intestinal detection status	
No suspected MP-like particles detected	27 (49.1%)
Suspected MP-like particles detected in ≥1 intestinal segment	28 (50.9%)
Segment-specific detection*	
Small intestine	12 (21.8%)
Large intestine	21 (38.2%)
Mutually exclusive detection pattern^†^	
Both intestinal segments	5 (9.1%)
Small intestine only	7 (12.7%)
Large intestine only	16 (29.1%)

Association between local histopathological alterations and suspected intestinal microplastic-like particle detection

Histological intestinal inflammation was associated with suspected intestinal MP-like particle detection, particularly when inflammation was present in the large intestine or in at least one intestinal segment (Figure 1). Small intestinal inflammation showed a weaker association with suspected MP-like particle detection that did not reach statistical significance. Histological evidence of intestinal necrosis was uncommon and showed a similar distribution between individuals with and without suspected intestinal MP-like particles. Given the small number of necrosis cases, these findings were interpreted descriptively, and no stable association with suspected MP-like particle detection could be established.

**Figure 1:**
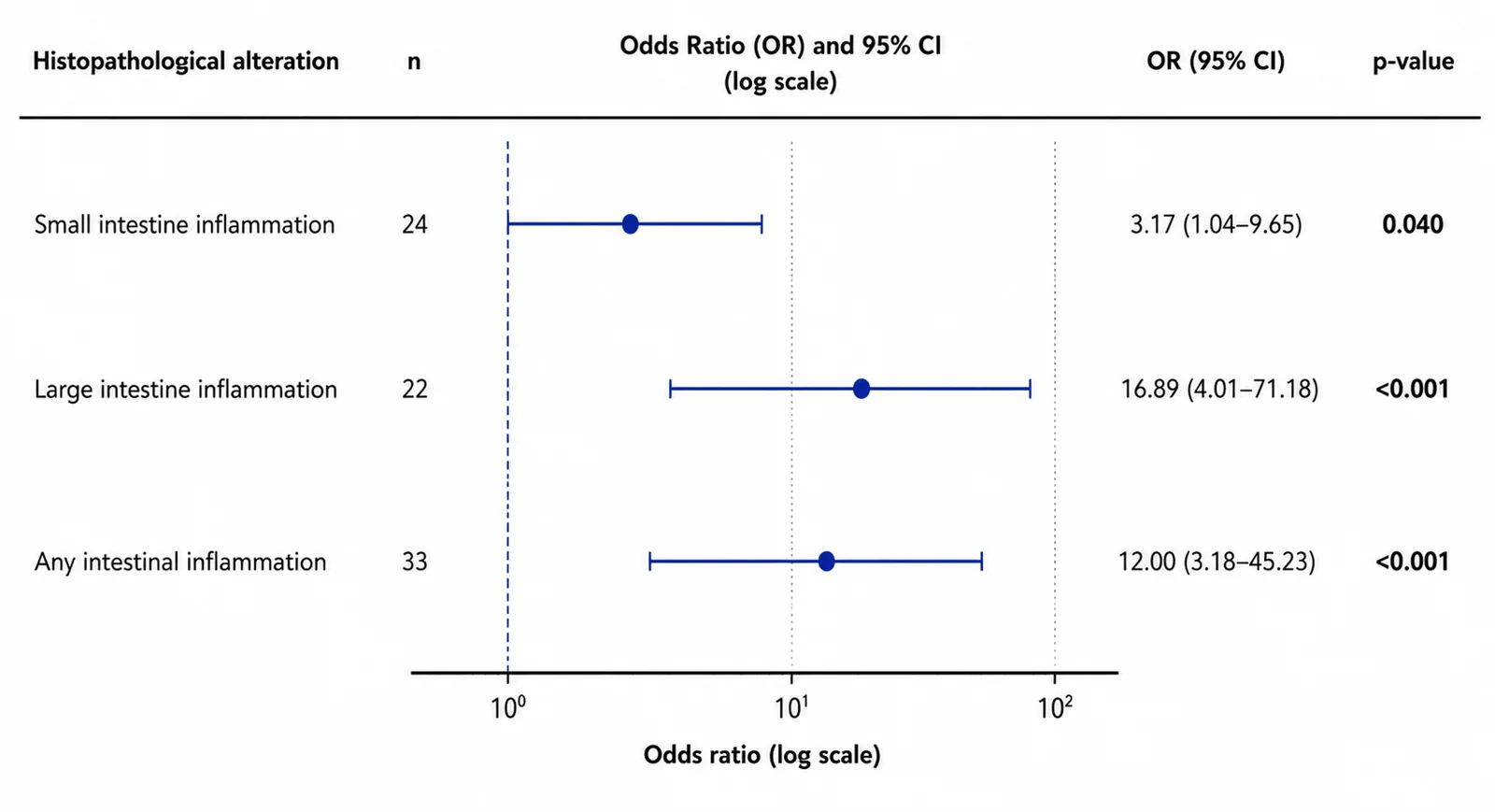
Unadjusted participant-level associations between histological intestinal inflammation and detection of suspected MP-like particles. The outcome was the detection of suspected MP-like particles in at least one intestinal specimen. For the segment-specific analyses, inflammation in the small or large intestine was compared with participant-level detection in either intestinal specimen and was not restricted to detection in the same intestinal segment. MP = microplastic; OR = odds ratio; CI = confidence interval

Intestinal fibrosis was not identified histologically in either intestinal segment; therefore, no inferential analysis was performed for this parameter. Overall, intestinal inflammation represented the clearest local histopathological finding associated with suspected intestinal MP-like particle detection, whereas necrosis was rare and fibrosis was absent in the analyzed samples.

Cardiopulmonary diagnoses in relation to suspected intestinal microplastic-like particle detection

Additional analyses were performed to evaluate the relationship between selected cardiovascular and pulmonary diagnoses and suspected intestinal MP-like particle detection. No statistically significant associations were observed for atherosclerosis, chronic ischemic heart disease, myocardial infarction, or bronchopneumonia (Figure 2).

**Figure 2:**
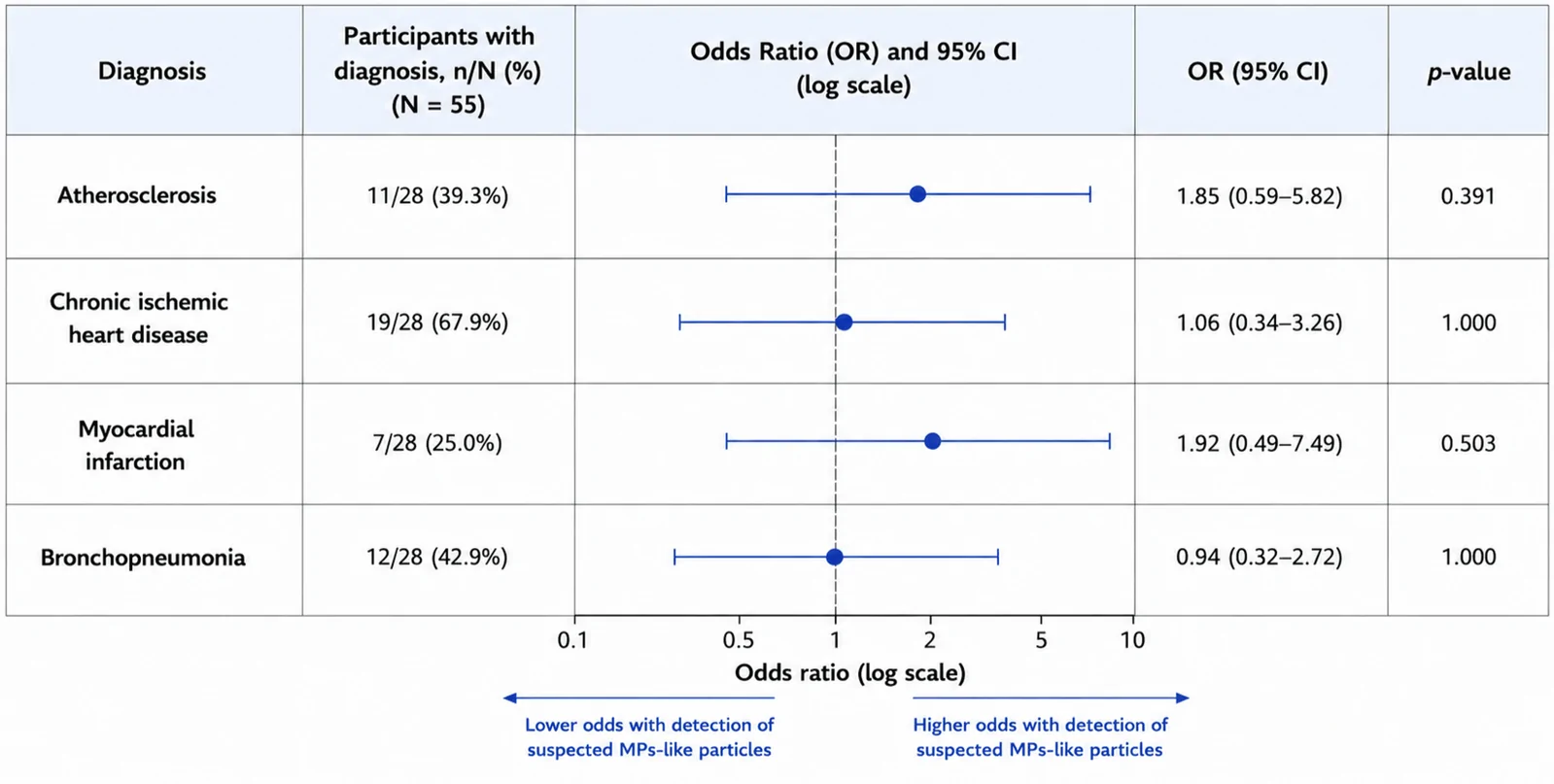
Cardiopulmonary diagnoses in relation to suspected intestinal MP-like particle detection. Suspected MP-like particles were detected in the small intestine, the large intestine, or both intestinal specimens. MP = microplastic; OR = odds ratio; CI = confidence interval

Suspected intestinal MP-like particles were numerically more frequent among individuals with atherosclerosis and myocardial infarction; however, these differences did not reach statistical significance. Detection rates were similar for chronic ischemic heart disease and bronchopneumonia. Overall, no clear cardiopulmonary diagnosis-specific pattern was identified in relation to suspected intestinal MP-like particle detection.

Polarized light visualization of selected suspected microplastic-like structures

Representative sections from small and large intestinal specimens were evaluated for suspected MP-like particles using polarized light microscopy as a complementary visual method (Figure 3). Exogenous particle detection was performed by analyzing paired polarized and unpolarized images from four selected cases. The images are not intended to estimate prevalence or validate polymer identity.

**Figure 3:**
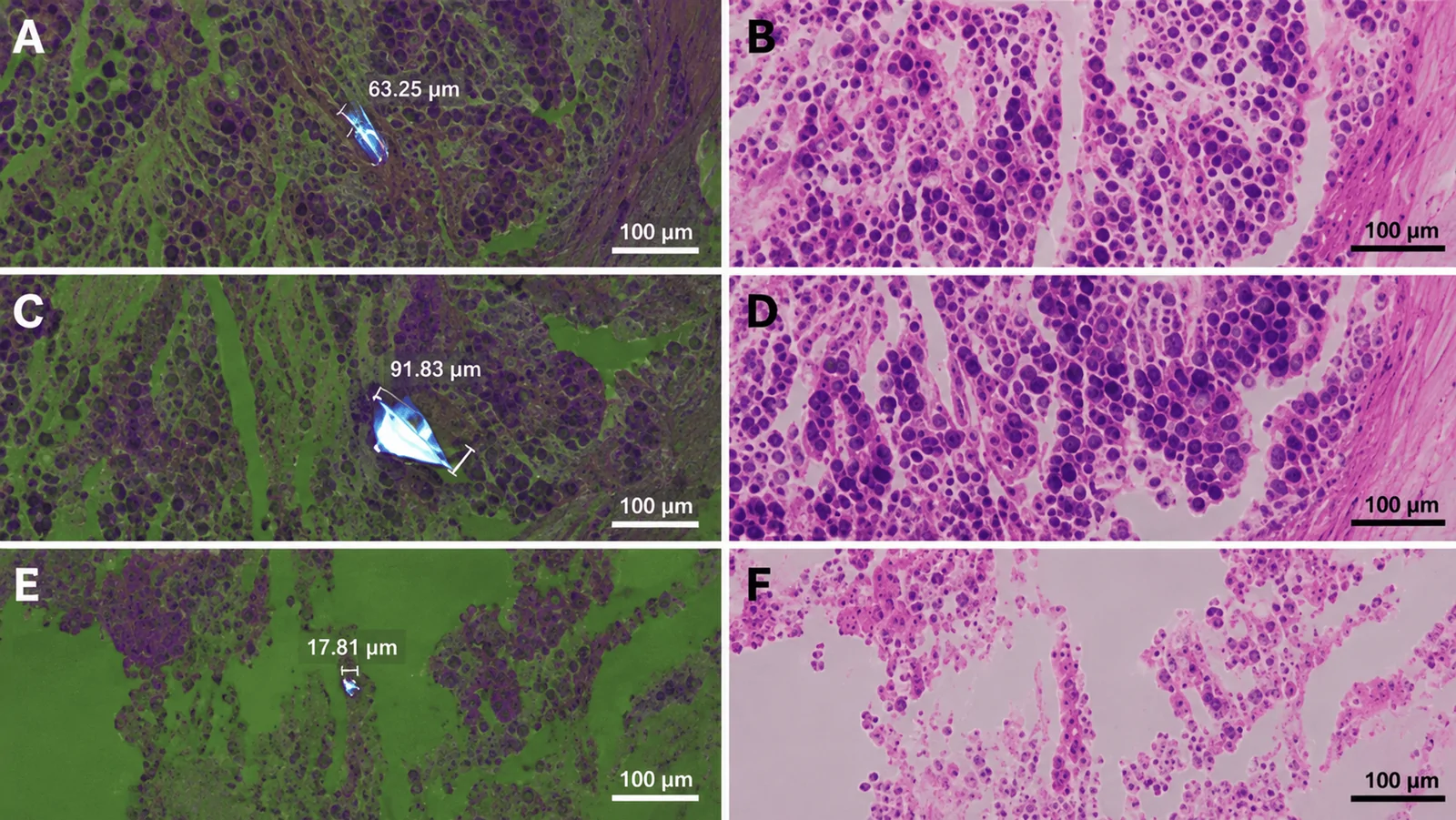
Representative polarized and unpolarized light microscopy images of suspected microplastic-like particles in small and large intestinal specimens. Measurement markers identify the structures of interest in the polarized panels. (A, B) Case 12, small intestine: 63.25 μm fibrillar structure with apparent intraepithelial localization. (C, D) Case 19, small intestine: 91.83 μm trapezoidal structure within the submucosa. (E, F) Case 22, large intestine: 17.81 μm luminal structure, which does not demonstrate tissue incorporation. These examples represent morphological screening observations; none underwent polymer-specific confirmation. Magnification: 40×. Scale bars: 100 μm.

Case 12 showed a 63.25 μm fibrillar birefringent structure with an apparent intraepithelial location. Case 19 showed a 91.83 μm trapezoidal birefringent structure within the submucosa. Case 22 showed a 17.81 μm ovoid luminal structure and therefore provided no evidence of tissue incorporation.

## Discussion

This exploratory post-mortem study identified specimen-level screening positivity for suspected MP-like particles in 28 of 55 individuals. Positivity was numerically more frequent in large than in small intestinal specimens, but the paired difference was not statistically significant. Because Nile Red fluorescence and polarized light birefringence are non-specific, the findings document candidate structures recovered using a screening workflow rather than confirmed MPs or tissue accumulation.

Ingestion and inhalation are plausible sources of gastrointestinal exposure to MP and NP particles, and experimental studies describe several potential routes of interaction with the intestinal mucosa [32-35]. These mechanisms provide biological context but cannot establish the origin, composition, uptake, or persistence of the structures observed in this study. Specimen-level recovery after digestion cannot distinguish residual luminal material, mucosal adherence, tissue incorporation, or contamination introduced during collection and processing.

Although large intestinal positivity was numerically more common, the data do not demonstrate preferential colonic accumulation. Slower distal transit and concentration of luminal contents through water absorption could increase the detectability of non-digestible material in large intestinal specimens [36,37]. These explanations remain speculative because transit time, stool burden, mucosal integrity, and exposure history were not measured, and polymer identity was not confirmed.

Screening positivity co-occurred with histological inflammation in unadjusted analyses. Experimental studies have reported oxidative stress, altered tight junction proteins, epithelial barrier dysfunction, and microbiome changes after microplastic exposure [38-40]. However, those experimental observations cannot establish causality in this cross-sectional post-mortem cohort. Pre-existing inflammation could facilitate adherence or trapping of exogenous material through epithelial disruption or altered mucus composition. Other explanations include underlying gastrointestinal or systemic disease, treatment, terminal illness, post-mortem changes, and contamination. Accordingly, the present findings do not demonstrate particle-induced inflammation, uptake, persistence, or accumulation.

Fibrosis was not identified, and necrosis was uncommon. The small number of necrotic lesions did not allow a reliable assessment of the relationship between suspected MP-like particle detection and structural tissue damage. Therefore, the predominance of inflammatory findings should not be interpreted as evidence that suspected particles cause inflammatory rather than structural injury [41].

No statistically significant differences were observed in systemic inflammatory biomarkers, hematological indices, hepatic, renal, pancreatic, or electrolyte parameters, or the examined cardiopulmonary diagnoses. Given the incomplete availability of data for some variables, the heterogeneity of the patients’ terminal clinical conditions, and the absence of an a priori power calculation for these secondary endpoints, these findings should be interpreted cautiously and cannot be used to confirm or exclude a systemic effect. Accordingly, the absence of statistically significant associations for these secondary outcomes should not be interpreted as evidence of no association, particularly given the exploratory design, limited sample size, variable data availability, and potential clinical confounding.

The urban-rural comparison produced an isolated p-value of 0.026, but exposure-relevant information (including diet, occupation, drinking-water source, socioeconomic status, and residential history) was unavailable. In view of multiple exploratory comparisons, this result may represent chance, residual confounding, or unmeasured exposure differences and requires independent confirmation.

The strengths of this study include paired small intestinal and large intestinal sampling and the integration of screening, histopathological, clinical, and laboratory observations. Nevertheless, the study remains exploratory because polymer identity was not confirmed, contamination monitoring was incomplete, the cohort was small and non-consecutive, and the analyses were predominantly unadjusted [42,43].

Limitations

This study has several limitations. The study population comprised a small, non-consecutive convenience sample of cases for which paired small and large intestinal specimens could be collected and processed according to the study protocol. Because a comprehensive autopsy screening log was not maintained, the total number and characteristics of cases not included are unknown. Selection bias therefore cannot be excluded, and the cohort may not be representative of the overall autopsy population. No a priori power calculation was performed for the secondary exploratory endpoints, the analyses were predominantly unadjusted, and no adjustment for multiple comparisons was applied.

Detailed exposure-related information, including diet, occupation, drinking water source, socioeconomic status, and residential history, was unavailable. Exact case-level post-mortem intervals, recent gastrointestinal procedures, and nutritional support were also not systematically available. Underlying gastrointestinal disorders were not used as specific exclusion criteria; therefore, inflammatory histological findings may also reflect pre-existing gastrointestinal disease and cannot be considered specific to suspected MP-like particle exposure. Clinical and laboratory data reflected the terminal hospitalization and were variably available across individuals, which further limits the interpretation of the observed associations and the ability to control for potential confounding.

The principal analytical limitation is that Nile Red fluorescence and polarized light microscopy are not polymer-specific; therefore, the detected structures should be interpreted as suspected MP-like particles rather than chemically confirmed microplastics. Although procedural reagent blanks and ABS recovery controls were used, separate airborne deposition, filter-only, transport, autopsy room, and histology processing blanks were not performed. Consequently, contamination introduced at these stages cannot be quantified or excluded. Furthermore, specimen-level recovery after digestion cannot distinguish residual luminal material, mucosal adherence, tissue incorporation, or contamination introduced during collection and processing.

## Conclusions

In this exploratory post-mortem study, suspected MP-like particles were detected in paired intestinal specimens using a non-polymer-specific screening method. Suspected MP-like particles were detected more frequently in large intestinal than in small intestinal specimens; however, the paired difference was not statistically significant. In unadjusted analyses, the detection of suspected MP-like particles was associated with non-specific histological inflammation. Nevertheless, polymer identity and tissue-compartment localization could not be confirmed, and the temporal sequence of this association or its independence from clinical and procedural confounders could not be established. These hypothesis-generating findings require confirmation in larger, clinically well-characterized cohorts using polymer-specific spectroscopic methods and comprehensive contamination controls. The present observations may help guide the design of future studies examining the anatomical distribution and potential biological relevance of suspected MP-like particles in the human intestine.
